# Giant Myxofibrosarcoma in the Lower Limb: An Overview of Diagnostic and Clinical Management

**DOI:** 10.3390/diagnostics14121298

**Published:** 2024-06-19

**Authors:** Răzvan Ene, Alexandru Lisias Dimitriu, Ileana Peride, Mirela Țigliș, Elisa Georgiana Popescu, Eduard Cătălin Georgescu, Tiberiu Paul Neagu, Ionel Alexandru Checherita, Andrei Niculae

**Affiliations:** 1 Clinical Department No. 14, “Carol Davila” University of Medicine and Pharmacy, 020021 Bucharest, Romania; 2 Clinical Department No. 3, “Carol Davila” University of Medicine and Pharmacy, 020021 Bucharest, Romania; niculaeandrei@yahoo.com; 3 Department of Anesthesia and Intensive Care, Emergency Clinical Hospital of Bucharest, 014461 Bucharest, Romania; 4 Clinical Department No. 11, “Carol Davila” University of Medicine and Pharmacy, 050474 Bucharest, Romania; dr.neagupaul@gmail.com; 5 Department of Nephrology, County Emergency Hospital Ilfov, 022104 Bucharest, Romania; al.checherita@gmail.com

**Keywords:** myxofibrosarcoma, lower limb, surgical resection, metastases, high-grade MFS, reconstructive surgery

## Abstract

Myxofibrosarcoma (MFS), an aggressive soft tissue sarcoma, is one of the undifferentiated pleomorphic sarcomas; it has a low incidence, affecting people in the sixth to eighth decades of life. It usually involves the extremities and is painless with a slow-growing pattern. Based on the case of a 52-year-old female patient who presented with a painful, massive, rapid-growing, ulcerated tumor of the anterior surface of the left thigh, we performed a literature review regarding the current standard of care for patients with MFS. Computed tomography examination, followed by magnetic resonance imaging and surgical biopsy with histopathological examination, confirmed the diagnosis and the presence of lung and inguinal lymph node metastases. Due to the rapid-growing pattern and the local aggressiveness, our tumor board team recommended emergency excisional surgery, with subsequent reconstructive procedures followed by referral to an oncological center. This review emphasizes the importance of proper and rapid diagnosis, followed by multidisciplinary management, for MFS cases with atypical presentation and distal metastases to improve overall outcomes.

## 1. Introduction

Myxofibrosarcoma (MFS), one of the undifferentiated pleomorphic sarcomas (20% of adult sarcomas), is known as an aggressive soft tissue sarcoma (STS) frequently located in subcutaneous and deep tissue areas [1,2]. According to the World Health Organization (WHO) Classification of Soft Tumors, MFS is part of the fibroblastic/myofibroblastic tumor group [3]. The incidence is about 0.1 per 100.000 per year (versus 2–5 per 100.00/year for all soft tissue sarcomas) [4,5]. Considering the histopathological heterogeneity in relation to the cellularity, nuclear pleomorphism, and proliferative pattern, it can be classified as low-, intermediate-, and high-grade myxofibrosarcoma [6]. The prognosis of MFS is good, with a 5-year survival rate of about 77%. Lower survival rates are reported in older patients and in cases with positive surgical margins, higher tumor grade, and larger size [7,8,9].

MFS commonly affects people in the sixth to eighth decades of life, having a slight predisposition in male patients. Although MFSs are the most common sarcomas, data about the incidence in relation to age group are scarce, with studies showing that cases under 20 years are extremely rare. Patient age has a reported median of between 42 and 66 years. The lower extremity is usually involved, followed by the upper extremity (combined, 77%), the trunk (12%), and the neck (3%), with other locations being rarely implicated [1,7,8,9,10,11]. It typically shows distant metastasis to the lungs, and rarely to the bones, lymph nodes, or liver [12]. MFSs located in soft tissues, mainly in the extremities, have a higher rate of local recurrence but a lower rate of metastasis compared with other sarcomas [13]. These tumors are normally painless with a slow-growing pattern and are classified as superficial (affecting the dermal and subcutaneous layers) and deep (involving muscular and subfascial tissues) [1,14].

Over the years, molecular studies have emphasized that MFS is one of the most intricate sarcomas regarding genomic complexity [15]. Immunohistochemical analyses showed that the expression of tumor endothelial marker 1 (TEM-1) is strong in MFS [16]. Smooth muscle actin (SMA), CD34, and muscle-specific actin (MSA) can be positive in cases of MFS [3]. Some molecules considered to be involved in MFS tumorigenesis are integrin-α10, mesenchymal–epithelial transition factor (MET) (patients with MET overexpression appear to have a higher risk of LR), neurofibromin-1 (NF1), ezrin, and melanoma-associated antigen 3 (MAGE-A3) [3,17,18,19,20,21]. MFS presents various important chromosomal aberrations, but none has high specificity [1].

Magnetic resonance imaging (MRI) is the imaging modality of choice in establishing the MFS diagnosis [7]. The standard of care is represented by surgical resection with negative margins before diagnostic confirmation through a biopsy, followed by radiotherapy or chemotherapy for intermediate- and high-grade sarcoma types or rapidly growing cases [22,23]. Complex vascular and plastic surgery reconstructive methods are often required for massive invasive tumors [8].

We performed a literature review based on the case of a female patient presenting a painful, giant, ulcerated myxofibrosarcoma of the left thigh, with a rapid-growing pattern and metastatic spread to the local lymph nodes and lung, which required rapid surgical resection followed by local reconstruction. The purpose of this review is to emphasize the current standard of care for patients with MFS, starting from clinical presentation, diagnosis, and classification, and to highlight the surgical management and the main adjuvant therapies, along with the tumor-associated morbidity and mortality, to improve the outcome and increase the survival of this subgroup of patients.

## 2. Case Presentation

**Figure 1:**
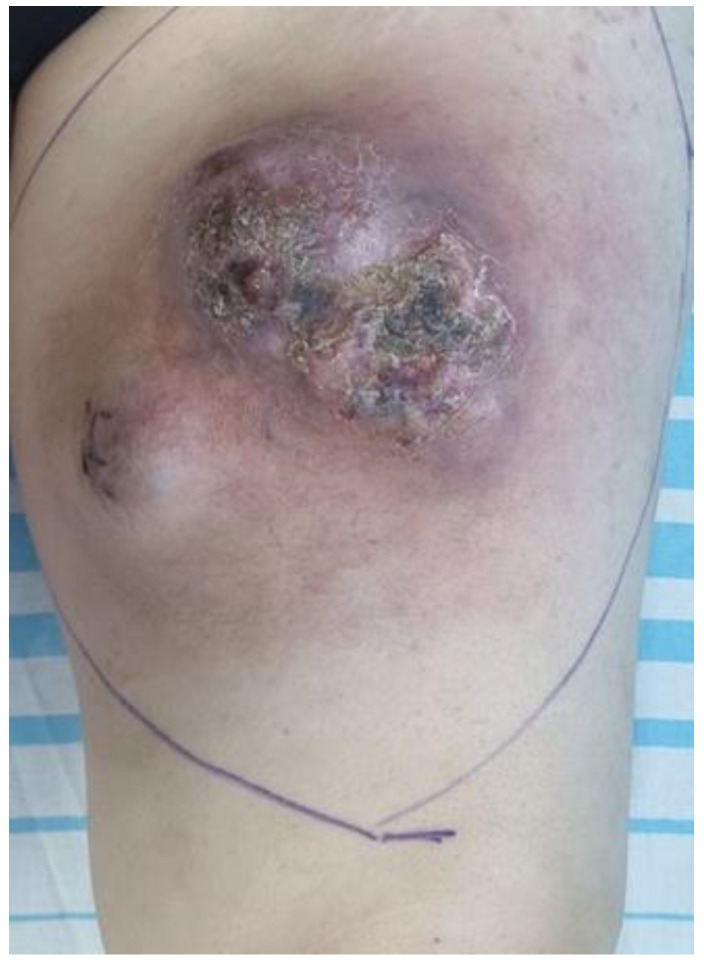
Clinical presentation of the myxofibrosarcoma—nodular aspect with crusted ulcerations, deep insertion affecting the anterior view of left thigh.

A 52-year-old female patient was referred to our hospital with a painful, massive, ulcerated tumor of the anterior left thigh following blunt trauma with a six-month rapid-growing pattern (Figure 1). Her past medical history included essential hypertension, second-degree obesity, and varicose veins of the left lower limb. She had no family history of MFS, but there was a recent local blunt trauma with subsequent hematoma formation. Clinical examination exposed a large, painful mass in the anterior left thigh, with multiple ulcerations and profound insertion. No signs of local infection were identified. All bacterial and fungal cultures were negative. The computed tomography (CT) examination revealed a well-defined pulmonary nodule of about 3 mm and left inguinal lymph node metastases. The tumor mass was in the medial part of the thigh, having a polylobate contour with well-defined margins of about 128 × 125 × 120 mm, but it showed signs of invasion in the subcutaneous tissue, with a posterior relationship to the femur but without bone invasion.

**Figure 2:**
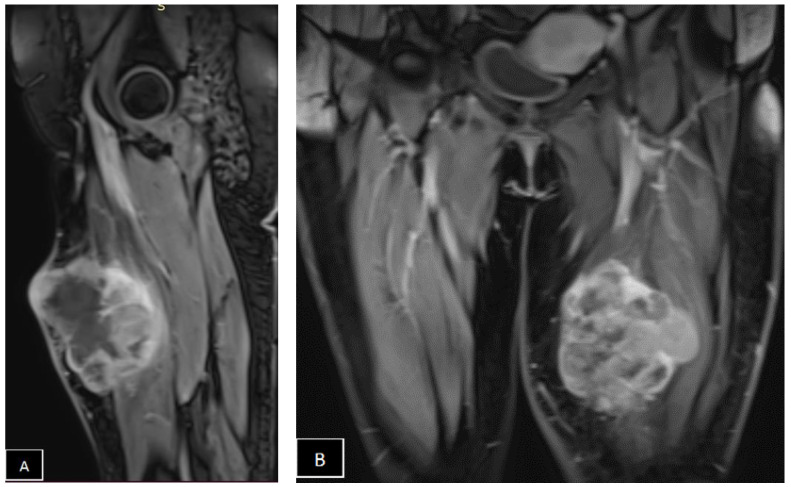

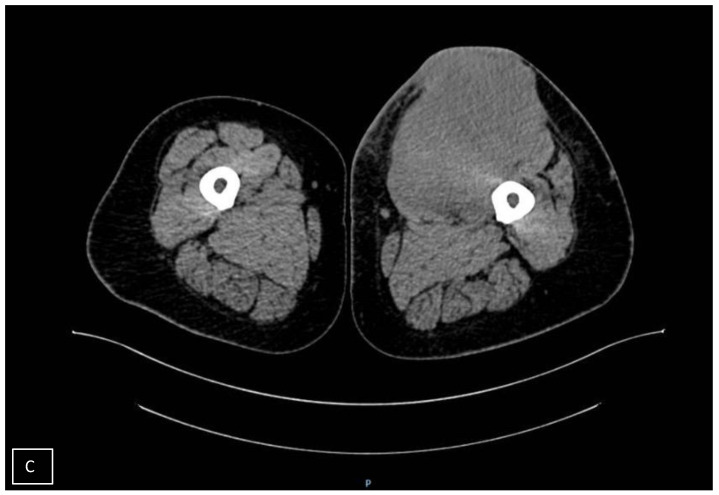
Nuclear magnetic resonance imaging (MRI) of the thighs. (**A**,**B**) Sagittal and coronal T1-weighted MRI. (**C**) Transverse T1-weighted MRI shows deep infiltration of the skin, the subcutaneous tissue, the left quadriceps, the vastus medialis, the vastus intermedius, and the rectus femoris muscles, and a highly intense irregular mass signal typical of the myxoid matrix in subcutaneous tissues, with an ill-defined margin in the upper third of the left thigh.

Afterwards, the MRI identified a heterogeneous mass of about 12 × 10.9 × 11.5 cm, probably with origins in the left quadriceps muscle, invading the vastus medialis, the vastus intermedius, and the rectus femoris muscles, fascia, and subcutaneous adjacent tissues, with features consistent with high-grade myxofibrosarcoma (Figure 2).

**Figure 3:**
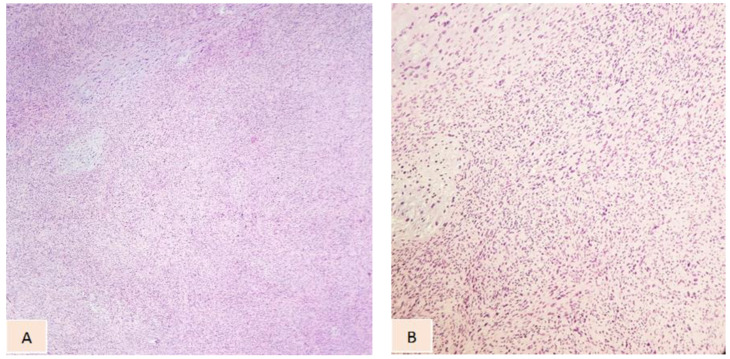
High-grade MFS: hypercellularity with diffuse fusiform cells, pleomorphic atypical cells, areas of necrosis, cells with variable density and multiple atypia, high mitosis, and myxoid background (H&E staining: (**A**) magnification ×100 and (**B**) magnification ×200).

Lower limb arteriography revealed that the tumor was highly vascularized, presenting collateral communication with the left femoral vein. A surgical biopsy followed by histological examination (microscopic and immunohistochemical analysis) confirmed the myxofibrosarcoma diagnosis, with microscopic analysis showing sarcomatous tumor proliferation, areas with loose myxoid stroma, necrosis, cells with pronounced cytologic atypia, and spindle-shaped and round–oval cells with variable density (Figure 3). Afterwards, the case was assessed by our tumor board team. Preoperative bioumoral evaluation showed mild leukocytosis, moderate hypochromic microcytic anemia, and mild thrombocytosis. Surgical intervention was required under general anesthesia; ellipsoid surgical resection of the tumor was performed, with negative margins (intraoperative rapid margin assessment), followed by femoral artery reconstruction with a synthetic graft and femoral vein reconstruction with an autograft from the ipsilateral saphenous vein. Coverage of the resulting defect was achieved using a myocutaneous pedicled rotated flap from the rectus abdominis muscle, with split-thickness skin grafts from the anteromedial surface of the calf and distal lateral aspect of the ipsilateral thigh. After the surgery, the patient was transferred to the intensive care unit (ICU) for further treatment and monitoring, with a favorable evolution. The patient maintained immobilization in a splint with knee flexion at 5 degrees for 3 weeks, being allowed to mobilize only with support. She was referred to an oncological center for postoperative chemotherapy. Postoperative local evolution was optimum at the one-month follow-up. Informed consent was obtained from the patient.

## 3. Discussion

Myxofibrosarcoma remains a difficult disease to diagnose and treat properly, with high morbidity; therefore, our recommendation for such a case, in accordance with the current standard of care, is to be referred to experienced centers where a multidisciplinary team can increase the likelihood of success.

### 3.1. Clinical Presentation

Patients with MFS usually present a painless mass that has a slow size increase, with 30–60% of tumors being deeply located [24,25]. Symptoms are normally related to the anatomical area of origin [3]. There are reported cases, like our patient, with a history of local trauma and hematoma, which makes the differential diagnosis difficult for several weeks [26,27,28]. Some studies suggest that ultrasound should be performed as a short-term follow-up, usually 3 to 6 weeks after the trauma, particularly to exclude a hematoma [26,29,30]. Klingbeil et al. presented the case of a male patient with chest wall MFS six months after a traumatic injury (scratched by his pet cat) [31].

MFS usually affects the extremities, as in our case, and rarely the trunk, the head, and the neck, and is initially asymptomatic [7,8,9,10]. There are rare cases of MFS in the brain, kidney, abdominal wall, larynx, and sinus piriformis [32,33,34,35]. Authors reporting the first primary renal myxofibrosarcoma warn about the importance of knowing the main clinical manifestations since MFS has a large spectrum of lesions with differential diagnoses and varying biologic behavior [34]. Bethel et al. reported the case of a giant myxofibrosarcoma involving the brachial plexus and causing compression of the airway structures [36]. An interesting case of MFS presented a Pancoast tumor-like manifestation with upper extremity pain, hand muscle atrophy, and Horner`s syndrome [37]. It can also present as pain in the knee joint [38]. There are rare reports of bilateral metachronous myxofibrosarcomas [39,40].

There are reported cases with paraneoplastic secretion of insulin-like growth factor (IGF) causing hypoglycemic attacks in patients with myxofibrosarcoma. In these patients, hypoglycemia ceased after the surgical removal of the tumor or after chemotherapy and radiotherapy [41,42].

### 3.2. Diagnosis and Classification

Ultrasonography (US) represents the first-line modality for evaluation, and it may be helpful in guiding percutaneous biopsies in specific cases [43]. CT and positron emission computed tomography (PET-CT) are usually used for metastasis detection and to appropriately identify the aggressive areas of the tumor to perform biopsies and restage recurrent cases [44,45]. MRI is preferred for preoperative evaluation, planning, and grading. MFS has specific MRI features like the tail sign (the infiltrative growth pattern), typical nodular or lobular lesions, hemorrhage, and necrosis (in high-grade tumors) [46,47]. For our case, after the computed tomography examination with high suspicion of MFS with distant metastases, MRI analysis, along with surgical biopsy and histopathological examination, allowed us to confirm the diagnosis and better understand the invasiveness of the tumor.

As we emphasized, MFSs are classified into two categories [1,7,10,48,49]:

▪ Superficial—lesions that affect the dermal and subcutaneous layers of the skin; these have an infiltrative pattern and are more frequent.
▪ Deep—lesions that affect the intramuscular or subfascial tissues, usually presenting as a single, nodular mass with a longitudinal pattern of spread.

Histologically, myxofibrosarcomas present with multinodular growth, incomplete septa, and myxoid stroma, and are classified into three grades [3,50,51]:

▪ Low-grade MFS (grade 1)—a low number of atypical spindle cells, variable myxoid matrix with hyperchromatic nuclei, rare mitotic activity, and no tumor necrosis.
▪ Intermediate-grade MFS (grade 2)—moderate cellularity and pleomorphism, rare solid areas, mild mitotic activity, and no tumor necrosis.
▪ High-grade MFS (grade 3)—high cellularity with intense pleomorphism, intense mitotic activity, atypical mitosis, prominent myxoid stroma, and tumor necrosis.

Tumor-related mortality and morbidity, and the risk of distant metastasis development, are related to the MFS histological grade [5]. The infiltrative pattern is responsible for local recurrences (LRs) and influences prognosis [52]. Furthermore, the presence of tumors > 5 cm with extensive necrosis (≥10%) and a high mitotic count (≥20/10 hpf) is a sign of aggressive behavior [25,35,53,54].

The main differential diagnosis is represented by but not limited to malignant peripheral nerve sheath tumors, myxoma, low-grade fibromyxoid sarcoma, pleiomorphic leiomyosarcomas, pleomorphic rhabdomyosarcomas, spindle cell lipoma, pleomorphic or dedifferentiated liposarcoma, melanoma, undifferentiated carcinoma, undifferentiated sarcoma, and nodular fasciitis [35,51,55].

### 3.3. Surgical and Adjuvant Management

Patients with MFS should be treated in specialized centers, with an interdisciplinary tumor board evaluation before surgery being essential to increase survival, as in our case [56,57]. Negative surgical margin resection (wide radical resection) is the standard of care for MFS, although it can be difficult considering the multidirectional spreading pattern of this tumor along the fascial planes. Resection and en bloc removal of the mass, followed by local tissue reconstruction (muscle flaps, skin grafts), are practiced for large tumors, as in our case [36,41,58]. It is recommended to aim for a resection margin of at least 1 cm, or, even better, a 2 cm width, to reduce the risk of LR [59,60]. MFS grade 2 and grade 3 tumors with positive surgical margins, and tumors originating in visceral organs (kidney, lungs), require surgery and adjuvant therapies [34,58].

In many cases of intermediate- and high-grade MFS, adjuvant chemotherapy and radiotherapy are usually required to prevent recurrence and improve outcomes, but this topic remains controversial [58,61]. In a study of 56 patients with MFS, Teurneau et al. showed that there was no difference with respect to LRs between those receiving and those not receiving radiotherapy [62]. In the case of giant MFS, preoperative radiotherapy may be performed to reduce the tumor mass [35]. Look Hong et al. reviewed the cases of 69 patients with MFS and emphasized that aggressive surgery along with radiotherapy may ensure effective local control by targeting the microscopic remains after tumor excision [63]. A study analyzing the cases of 158 patients with localized myxofibrosarcoma stated that the real value of chemotherapy in management remains undetermined [7]. Sambri et al. showed that radiotherapy may prevent LR development in the first 5 years after surgery [52]. For invasive tumors of the head and neck, when complete surgical resection is not possible, radiochemotherapy may prevent recurrence [37,64]. This combined therapy may ensure prolonged life expectancy compared with radiotherapy alone [35]. Combining surgery with adjuvant radiotherapy, in association with a regular follow-up schedule, is absolutely recommended by some authors to prevent local or distant metastases [65]. For our patient, the tumor board team decided in favor of rapid surgery, followed by a referral to an oncological center for postoperative chemotherapy.

Immunotherapy has been used for patients with MFS, especially for advanced cases, high-grade MFS, and those with distant metastases, showing the benefits of combination therapies by improving tumor response [66,67,68].

High-intensity focused ultrasound (HIFU) is an alternative therapy able to target tissues or tumors in a low-power cumulative way, and it showed beneficial effects in the case of recurrent MFS [69].

### 3.4. Prognostic Factors, Tumor-Associated Morbidity, and Mortality

Studies have shown that MFS, irrespective of surgical margin resection status, location, size, depth, or grade, leads to LRs in 22–79% of cases [70,71]. This appears to be related to the infiltrative growth pattern, i.e., tumoral cells spreading along fascial and vascular planes [72]. A study including 75 cases of myxofibrosarcoma showed that the depth of the primary lesion is not related to the incidence of local recurrence. However, deeply located masses are usually larger, high-grade, and infiltrative, and associated with a worse prognosis [1]. It appears that systemic inflammation (high level of serum C-reactive protein and high neutrophil–lymphocyte ratio) in the presence of high-grade MFS of the extremities leads to a worse prognosis [73].

Over the years, various factors have been implicated in the recurrence rate of MFS, such as tumor size and location, patient age, the myxoid component, and inadvertent excision during the first surgery [41,70,74]. We consider that one of the most important factors that influence the prognosis of these patients remains the capability to obtain negative surgical margins at first excision [41,74,75]. Dadrass et al. showed, in a retrospective study involving 42 surgically treated patients with MFS in a specialized center, that negative surgical margins were associated with a higher rate of LR; therefore, their recommendation was to favor larger negative margins [76]. Tumors larger than 5 cm and the presence of intralesional surgical margins are associated with distant metastasis development [62].

Considering the histopathological heterogeneity of this disease, Haglund et al. emphasized that, for intermediate- and high-grade infiltrative cases, despite aggressive negative-margin surgery followed by radiation, the risk of LR is increased, which is associated with high morbidity [62]. High-grade MFSs usually have an infiltrative growth pattern and specific histopathological characteristics, such as tumor necrosis, high cellularity, and severe nuclear atypia [6,36]. Lin et al. also showed that positive surgical margins, tumor necrosis, and the presence of mitoses are associated with poor outcomes [77]. In a series of 229 patients with MFS of the extremities, the authors indicated that grade 3 cases have the worst prognosis, with grade 1 patients having the lowest risk of LRs [78].

Metastatic MFSs are associated with poor prognosis and represent the main cause of death in patients with sarcomas [1,79]. Whereas low-grade myxofibrosarcomas have no metastatic risk, intermediate- and high-grade types are associated with metastasis development in 16–38% of cases [63,80,81]. Lung metastasis appears to have a shorter onset time compared with lymph node metastasis, is more aggressive, and has a lower survival rate [81]. It is considered that LRs, within 12 months after surgery, have a higher mortality rate [1]. Van der Horst et al. showed in a recent analysis that the median overall survival for patients with MFS was about 155 months, with a 5-year overall survival of 67.7% [82].

Considering that overall survival is influenced by age, tumor size, location, and extension, as well as surgical intervention, the need for chemotherapy and radiotherapy, and the number of tumors, Cao et al. developed a novel nomogram to evaluate the survival outcomes for patients with MFS based on tumor characteristics and treatment methods [83].

## 4. Conclusions

Myxofibrosarcomas, especially those with deep infiltration, remain a challenge in daily practice due to their unpredictable clinical evolution; therefore, they should be treated in specialized centers by multidisciplinary teams. The current standard of care involves performing a proper imaging diagnosis and histopathological analysis and ensuring tumor-free surgical margins after local excision. For extensive cases, such as our patient, a multidisciplinary team approach is essential to ensure a high curative rate, with proper local tissue reconstruction to provide the maximum functionality and quality of life and a low LR rate.
